# Exploring Migratory Grief in Teenagers: A Case Report

**DOI:** 10.1192/j.eurpsy.2026.11633

**Published:** 2026-07-31

**Authors:** D. A. Sanchez Herrera

**Affiliations:** Psychiatry, Hospital Universitario Virgen de la Victoria, Malaga, Spain

## Abstract

**Introduction:**

Migration is a complex social, cultural, and psychological phenomenon that has increased significantly in recent decades. While migration can provide opportunities for personal and family development, it also entails multiple losses: home, language, social ties, routines, and cultural identity. These changes may trigger a process known as migratory grief, which is distinct from pathological grief but can become clinically relevant when adaptation is impaired. Adolescents are particularly vulnerable to migratory grief. At this stage of life, identity formation, social integration, and autonomy are crucial developmental tasks. Disruption of these processes by migration may result in maladaptive coping strategies.

**Objectives:**

The objectives were to examine the literature and to present a clinical case of migratory grief focusing on clinical presentation, intervention, and short-term outcomes.

**Methods:**

A review was conducted using major sources of medical and non-medical information. In addition, the clinical experience of the team in this field was incorporated.

**Results:**

The patient is a 17-year-old male who presented to the emergency department accompanied by his parents due to episodes of verbal and physical aggression directed both at himself and at family members. He has no previous history of psychiatric treatment. Originally from Colombia, he moved to Spain last year with his family. According to his parents, during the past two months, he began experiencing anxiety crises at home, accompanied by behavioral disturbances characterized by screaming, physical aggression, property destruction, and an episode of running away from home. In the most recent episode, he went to his grandparents’ house, where he began destroying objects, displayed verbal aggression toward them, and engaged in self-directed physical aggression. For this reason, his parents called the emergency services and the police. Upon arrival at the emergency department, his behavioral symptoms and anxiety could not be controlled, which led to hospitalization. He reports poor adaptation to his current environment, absence of friendships, school dropout, limited social interactions outside his family, refusal of unfamiliar foods, and avoidance of contact with people other than close relatives. During hospitalization, the patient showed a reduction in anxiety levels, no further crisis episodes occurred at home, and he began to establish new relationships.

**Conclusions:**

This case demonstrates how migration can profoundly affect adolescent mental health, with migratory grief serving as a central explanatory framework. Adolescents, in the midst of identity formation and social integration, are especially vulnerable to the cumulative losses associated with migration. Recognition of this phenomenon is essential for mental health professionals, as symptoms may otherwise be misattributed to primary psychiatric disorders without consideration of the psychosocial context.

**Disclosure of Interest:**

None Declared

